# An Equilibrium Strategy-Based Routing Optimization Algorithm for Wireless Sensor Networks

**DOI:** 10.3390/s18103477

**Published:** 2018-10-16

**Authors:** Liangrui Tang, Zhilin Lu, Jinqi Cai, Jiangyu Yan

**Affiliations:** State Key Laboratory of Alternate Electrical Power System with Renewable Energy Sources, North China Electric Power University, Beijing 102206, China; tlr@ncepu.edu.cn (L.T.); 1172201352@ncepu.edu.cn (J.C.); yabjy@ncepu.edu.cn (J.Y.)

**Keywords:** wireless sensor networks, “edge-cutting” strategy, load balancing, energy saving, routing

## Abstract

In energy-constrained wireless sensor networks (WSNs), the design of an energy-efficient smart strategy is a key to extend the network lifetime, but the unbalance of energy consumption and node load severely restrict the long-term operation of the network. To address these issues, a novel routing algorithm which considers both energy saving and load balancing is proposed in this paper. First of all, the transmission energy consumption, node residual energy and path hops are considered to create the link cost, and then a minimum routing graph is generated based on the link cost. Finally, in order to ensure the balance of traffic and residual energy of each node in the network, an “edge-cutting” strategy is proposed to optimize the minimum routing graph and turn it into a minimum routing tree. The simulation results show that, the proposed algorithm not only can balance the network load and prolong the lifetime of network, but meet the needs of delay and packet loss rate.

## 1. Introduction

### 1.1. Background and Motivation

With the acceleration of the Internet of Things era, wireless sensor networks (WSNs) have been widely deployed in various application scenarios, such as volcanic eruption monitoring [1,2], medical care monitoring [3], and smart home monitoring [4]. Therefore, many studies about the topic have been done, including load balancing, security, QoS, congestion control, connectivity, coverage [5,6,7,8,9,10,11], etc. Besides, due to the limited energy storage [12,13] of battery-powered sensor networks, these applications also have a common requirement for the network lifetime, thus reducing the energy consumption to extend the network lifetime is one of the key tasks of the WSN. However, some nodes undertake excessive data forwarding because of the location factors, and the network is prone to energy hole phenomenon due to uneven energy consumption [14], which resulting in faster node death and lower network lifetime. Therefore, in order to maximize the network lifetime, both energy consumption reducing and load balancing are needed to be considered when making routing decisions.

### 1.2. Related Works

In some traditional routing algorithms that extend the network lifetime by reducing network energy consumption [15,16], the energy consumption is saved mainly by reducing the hop counts of the transmission paths. Among them, the GBR algorithm [15] finds the path with the least hops from source node to the sink by calculating the parameter “node height”; Min-Hop [16] chooses a node among the neighbors as the relay, which has the least hops from source node to the sink node, and when there are multiple paths with the fewest hop counts, the residual energy of the nodes will be taken as a decisive factor. This type of algorithms based on the minimum hops is equivalent to the minimum energy consumption routing [17]. Generally, it can reduce the path energy consumption, but it is easy to cause some nodes to exhaust prematurely due to overuse, bringing about an uneven distribution of residual energy and a short network lifetime. In addition, there are some routing algorithms that consider the sleep strategies [18,19,20]. These algorithms lessen the energy consumption rate by adjusting the “sleeping” and “awake” working modes of nodes, thus prolonging the lifetime of the entire network. However, there always exists a trade-off between energy efficiency and other performances.

For the above problems, some routing algorithms that consider the load balancing are proposed in [21,22,23,24,25,26]. Among them, SLDD [21] uses the evaluation function to select a super-link to redistribute network load through nodes with large energy and communication capacity on the super-link. However, since only a single metric is considered in the routing decision, this strategy can only be locally optimal, which may make other nodes exceed the load capacity [27]. In order to avoid the nodes in hot spot from taking too many forwarding tasks, in [22], the transmission power is controlled to balance the load, which can shrink the difference of residual energy of each node and achieve the purpose of extending the network lifetime, but for the network performance optimization, such as the network throughput and transmission delay, it is irrationally overlooked. DECOR [23] proposes a degree-limited routing, in which the load-balanced routing tree is established by limiting the degree of each node, but the required overhead of the computation and communication in this algorithm are large. To ensure the load balancing of node communication, a heuristic multi-path algorithm is proposed in [24], but it ignores the energy consumption caused by the distance factor. In [25], the FAF-EBRM adopts an energy balancing routing protocol based on a forward sensing factor, and utilizes the energy density of the forward region and the link data traffic to make routing decisions, which can alleviate data congestion in some way, similarly, due to the energy loss of detours, it is hard to minimize the path energy consumption. In [26], FLEOR can reduce the energy waste caused by the detours on the basis of the fuzzy theory, it selects the next hop by considering the transmission distance, hop counts and energy balance of the path, which can reduce the transmission energy consumption and prioritize the nodes with higher residual energy. Nevertheless, the fuzzy rules are formulated artificially, which lead to the lack of objectivity of next hop selecting, so the final path may not be the optimal. 

Therefore, in order to maximize the network lifetime, we both consider reducing the energy consumption of the whole network and balancing the node load, proposing an equilibrium strategy-based routing optimization algorithm for wireless sensor networks (ESRA). 

### 1.3. Contributions

Based on the above analysis, the major contributions of this paper are summarized as follows:

- We defined the link cost for the sake of energy saving and less delay, by considering the residual energy of the node, the transmission energy consumption and the forward energy consumption of the next hop.
- We generated the minimum routing graph based on the link cost. Here, in order to obtain this graph, the shortest path set of each node is calculated from the source node to the sink node according to the improved Dijkstra algorithm [28].
- We proposed an “edge-cutting” strategy to balance the load in the minimum routing graph, so that the network structure can be adjusted in real time to optimize the route by dynamically sensing the node load. Simulation results show that our algorithm can decrease the average network energy, balance the node load, extend network lifetime, and also reduce transmission delay and packet loss rate, showing a good network performance.

The rest of this paper is organized as follows: in Section 2, we describe the network model and related definitions. Section 3 introduces the proposed ESRA algorithm in detail. The performance of our algorithm is analyzed and discussed in Section 4 according to relevant simulation results. Finally, the conclusions drawn are summarized in Section 5. 

## 2. Network Model and Related Definitions

### 2.1. Network Model

In this paper, we focus on the typical information collection application in event-driven wireless sensor networks. The sensor nodes periodically perceive the data and transmit them to the sink node in a multi-hop manner. In order to simplify the network, it is assumed that:

(1) All sensor nodes are isomorphic and randomly deployed in a certain monitoring area. The sensor nodes have only one sink node, and the location of sensor nodes and the sink node will not be changed after being deployed.
(2) The node can change the transmission power according to the distance to the receiver, and the distance from one sensor node to another can be estimated based on the received signal strength.
(3) In a practical application, the packet size and the data generation rate can be determined according to different scenarios. As for this paper, in order to simplify the model, the size of the packet is fixed and the data generation rate is the same for all nodes.

### 2.2. Related Definitions

In order to describe our algorithm more clearly, the relevant definitions are provided as follows:

**Definition** **1.** *Network lifetime: the round of the first dead node due to energy exhaustion [29,30,31,32,33].*

**Definition** **2.** *Forward energy consumption: the distance from the node* i *to the sink is defined as the forward distance* $d_{is}$ *, thus the forward energy consumption* $e_{is}$ *can be calculated according to the forward distance* $d_{is}$ *and the energy consumption model in [34]. Namely, the model is expressed as:*

(1) $$\begin{array}{ccc} E_{t}(\lambda ,d) & =\{\begin{array}{c} \lambda E_{bas}+\lambda \varepsilon_{fs}d^{2}\\ \lambda E_{bas}+\lambda \varepsilon_{mp}d^{4} \end{array} & \begin{array}{c} d<d_{0}\\ d\geq d_{0} \end{array} \end{array}$$

(2) $$E_{r}(\lambda )=E_{bas}(\lambda )=\lambda E_{bas}$$

*where* λ *represents the length of the packet, and* $\varepsilon_{fs}$ *,* $\varepsilon_{mp}$ *represent the transmit amplifier, d is the distance of node sending data,* $d_{0}=\sqrt{\varepsilon_{fs}/\varepsilon_{mp}}$ *denotes the threshold of the communication distance,* $E_{bas}$ *is the energy consumed when transmitting or receiving 1 bit of data. Here, considering that the receiving energy consumption* $E_{r}(\lambda )$ *is much smaller than the sending energy consumption* $E_{t}(\lambda ,d)$ *, so we ignore it in the calculation.*

**Definition** **3.** *Node load: under the assumption that the data generation rate of all nodes in the network is the same, the number of descendant nodes of the node* i *reflects the load size in some way. At the same time, it is considered that the node itself also generate monitoring data, so the node load* LN(i) *is defined as the number of all nodes (namely descendant nodes) that can transfer data to node i and also plus itself.*

**Definition** **4.** *The candidate parent set: in the generated minimum routing graph, all parent nodes on the multiple shortest paths of node i are defined as candidate parent set*CN(i).

**Definition** **5.** *The adjacent node set: a set of nodes, whose nodes are one hop away from the nodes on the initial shortest path of node i and can transmit data to them, are denoted as* NBS(i) *.*

**Definition** **6.** *Transmission energy refers to the energy consumed by the source node sending the data to next hop, and the path energy consumption represents the sum of the transmission energy of each node on the path.*

## 3. ESRA Routing Algorithm

In a WSN, in order to improve the energy efficiency of the entire network and maintain a long network lifetime, it is usually necessary to adopt an energy-efficient routing strategy to reduce energy consumption. At the same time, the node load can not only reflect the traffic of the node, but also predict the residual energy of a node to some extent because of the transmission energy consumption generated by forwarding data. Therefore, so as to avoid the uneven energy distribution caused by too light or too heavy loads of the nodes, the load balancing of the nodes also should be considered.

Firstly, our algorithm considers the two factors of energy consumption and hops to establish the link cost, and thus generates a minimum routing graph. Secondly, in order to balance the load of nodes, an edge-cutting strategy is implemented on the minimum routing graph. Among this process, a candidate parent node with the smallest load is set as next hop, and the links between source node and other candidate parent nodes are removed, thus the minimum routing tree is obtained so as to avoid the load unbalance caused by the influx of large amounts of data. The data flow is transmitted along the minimum routing tree, which will be dynamically adjusted by sensing the load of all sensor nodes and updating their residual energy in real time.

### 3.1. Link Cost

Due to the limited communication distance of the sensor nodes, the nodes generally need to transmit the sensed data to the base station in a multi-hop manner. Therefore, how to establish a path with the least energy consumption from source node to the sink node is a priority for the routing algorithm. Here, in order to avoid data backhaul and ensure that data are transmitted forward along the direction of the sink, according to the model of [25], the forward neighbor node set of node *i* is defined as follows:

(3) $$FN(i)=\{j|d_{ij}\leq R,d_{js}<d_{is}\}$$

where $d_{ij}$ is the distance from node *i* to node *j*, $d_{is}$ and $d_{js}$ are the distance from node *i* and node *j* to sink respectively, *R* represents the maximum communication radius of node *i*.

**Figure 1:**
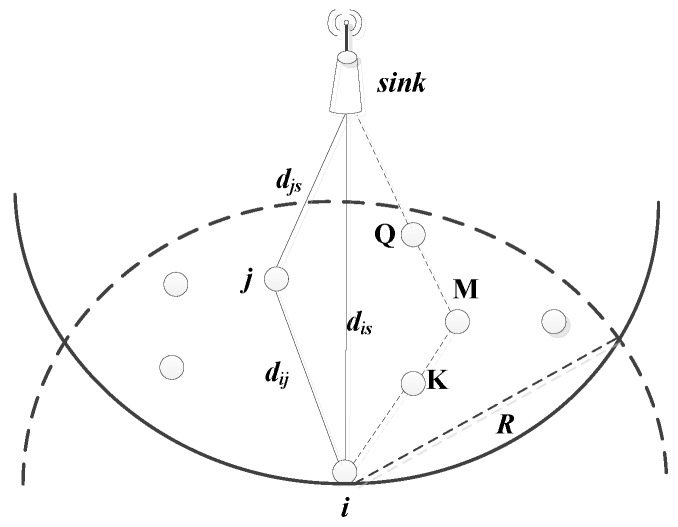
Spatial positional relationship between nodes.

As shown in Figure 1, after taking the spatial positional relationship between the current node *i*, the next hop *j*, and the sink node, as well as the energy level of each node into consideration, a data transmission path reducing energy consumption and delay is established in this paper. For source node *i*, the smaller the energy consumption of the single-hop transmission is to next hop node *j*, the slower the energy consumption caused by the data transmission. But for the overall path, if the path with a small single-hop distance (that is, the transmission energy consumption is small) is selected multiple times, there may exist more unnecessary energy loss and larger path hops will be caused due to the detour (such as the path: i→K→M→Q→sink). Therefore, in the process of generating the link cost, the forward distance of next hop *j* should also be considered, because the closer the next hop is to the sink, and the less path hops it will be, thus the faster that data can be transmitted to the sink. Based on the above analysis, in order to reduce the energy consumption and transmission delay of node *i*, the path where the next hop is located should be as close as possible to the straight line of node *i* to the sink node.

Therefore, in this paper, from the perspective of energy consumption and hop count, we take into account the residual energy, the energy consumption of single-hop transmission, and the forward energy consumption, the link cost $u_{i,j}$ between node *i* and node *j* is defined, and its expression is as follows:

(4) $$u_{i,j}=\alpha \frac{e_{ij}}{E_{i}}+\beta \frac{e_{js}}{E_{j}},j\in FN(i)$$

where *j* belongs to the forward neighbor node set of current node *i* to avoid the return of data packets, $e_{ij}$ is the transmission energy consumption from node *i* to node *j*, $e_{js}$ is the forward energy consumption from node *j* to sink, and both of them can be calculated by the energy consumption model in [34]. $E_{i}$, $E_{j}$ are the current residual energy of node *i* and node *j* respectively. Besides, it should be noted that the link cost is set to infinity when the distance between node *i* and node *j* is longer than the communication radius *R*.

Simultaneously, in Equation (4), α, β are the positive harmonic coefficients, and satisfy α+β=1. When the value of α is larger, the selection of next hop tends to consider the residual energy of the current node and the transmission energy consumption of a single hop, seeking to minimize the energy consumption of transmission and maximize the residual energy, so that the ratio of the two is as small as possible. Conversely, when the value of α is smaller, the residual energy and forward energy consumption of forward node are considered to select the next hop, trying to make the ratio of the two as small as possible to reduce the hop counts, so that the data can be transmitted to sink faster. Therefore, by adjusting the relative values of α and β, different requirements of network performance in hop counts and energy consumption can be obtained.

According to Equation (4), when the network size is *N*, the link cost matrix of the whole network can be expressed as:

(5) $$U=\left[\begin{array}{lllll} u_{1,1} & u_{1,2} & \cdot \cdot \cdot & u_{1,N} & u_{1,s}\\ u_{2,1} & u_{2,2} & \cdot \cdot \cdot & u_{2,N} & u_{2,s}\\ \vdots & \vdots & \ddots & \vdots & \vdots \\ u_{N,1} & u_{N,2} & \cdot \cdot \cdot & u_{N,N} & u_{N,s} \end{array}\right]$$

### 3.2. Generation of Minimum Routing Graph

Using the link cost in Section 3.1 as a weight, all shortest paths from any node *i* to sink can be found to form the minimum routing graph. Specifically, as it has been shown in Algorithm 1, the Dijkstra algorithm can be used to obtain an initial shortest path from node *i* to the sink, which is recorded as $mp_{0}^{is}$ and added to the shortest path set $MP^{is}$ of node *i*. At the same time, it can be known from [28] that those nodes on the other shortest paths must be distributed around the initial shortest path $mp_{0}^{is}$. Therefore, the adjacent nodes in NBS(i) and the nodes in the shortest path $mp_{0}^{is}$ can be chosen to form n candidate paths $path_{t}^{is}(1\leq t\leq n)$ from node *i* to the sink, and these paths are recorded as $P^{is}$. It should be noted that when *t* = 1, $path_{t}^{is}$ is set as the initial shortest path $mp_{0}^{is}$.

Here, the total link cost of any path $path_{t}^{is}$ is defined as follows:

(6) $$w_{t}^{is}=\sum_{j=1}^{j=|path_{t}^{is}|-1}u_{j,j+1}$$

Thus, the total link cost $w_{t}^{is}$ of all candidate paths $path_{t}^{is}$ in the set $P^{is}$ can be sequentially calculated according to Equation (6), and when $w_{t}^{is}$ is equal to the total link cost of the initial shortest path $w_{1}^{is}$, this candidate shortest path will be added to $MP^{is}$.

**Algorithm 1** The Generation of Minimum Routing Graph**Input:** the link cost matrix U, the network size *N* **Output:** the shortest path set $MP^{is}$ 1. **for**
*I* = 1:*N* 2.  Calculate the initial shortest path $mp_{0}^{is}$ of node *i* by Dijkstra algorithm. 3.  **update**
$MP^{is}\leftarrow MP^{is}+mp_{0}^{is}$ 4.  Find the set of adjacent nodes NBS(i) of the shortest path $mp_{0}^{is}$. 5.  *k* = 1   // Start finding adjacent nodes from the first node of $mp_{0}^{is}$. 6. **while** ($k\;<\;|mp_{0}^{is}|$) 7.   Insert adjacent nodes from the *k*th node of $mp_{0}^{is}$, and get the candidate path $path_{t}^{is}$ under the current *k*. 8.   **update**
$P^{is}\leftarrow P^{is}+path_{t}^{is}$ 9.   *k* = *k* + 1 10. **end**    // Get the candidate path set $P^{is}$. 11. *g* = 2 12. **while** (g≤n) 13.  Calculate the link cost $w_{g}^{is}$ by Equation (6). 14.  **if**
$w_{g}^{is}=w_{1}^{is}$ 15.    **update**
$MP^{is}\leftarrow MP^{is}+path_{g}^{is}$ 16.  **end** 17.  *g* = *g* + 1 18. **end** 19. **end** 20. ***return*** the shortest path set $MP^{is}$

### 3.3. Path Optimization Based on Edge-Cutting Strategy

If the data are transmitted along the minimum routing graph, the average energy consumption and hops of the whole network can be effectively reduced. However, due to ignoring the loads of nodes, this routing strategy will easily lead to load unbalance and large differences of residual energy between nodes, thus reducing the network lifetime. 

Therefore, as it is shown in Algorithm 2, this section proposes an edge-cutting strategy to “trim” the minimum routing graph. In this edge-cutting strategy, in order to avoid a large amount of data influx into some nodes at the same period, all nodes in the minimum routing graph dynamically select the candidate parent nodes with the smallest load as the next hop, which can promote the balance of energy and traffic of each node. Furthermore, it can improve energy efficiency and prolong network lifetime simultaneously.

As it can be seen from Section 3.2, a node may have multiple shortest paths. In order to make each node evenly undertake data forwarding tasks and avoid node congestion, the candidate parent node with smaller node load should be selected as the next hop as much as possible, and dynamically adjust it as the load changes.

**Algorithm 2** The Edge-Cutting Strategy**Input:** the minimum routing graph, the layered set *L*, initial load LN(i) of node *i* in the minimum routing graph **Output:** the minimum routing tree 1. **for**
k=1:(|L|−1) 2.  **while** ($|l_{k}|\;>\;0$)  //complete edge-cutting for all nodes of layer $l_{k}$. 3.   **if** exist a node “*a*” with the least load at the layer $l_{k}$ 4.    **if** exist multiple candidate parent nodes of the child nodes of node “*a*” 5.     Cut off the link between the child nodes of “*a*” and other candidate parent nodes. 6.    **end** 7.   **else if** exist multiple nodes with the least load at the layer $l_{k}$ 8.      Calculate the node product LCN(i), and set the node with the smallest node product to “*a*”. 9.     **if** the child node of node “*a*” exist multiple candidate parent nodes. 10.       Cut off the link between the child nodes of “*a*” and its other candidate parent nodes. 11.     **end** 12.   **update**
$l_{k}\leftarrow l_{k}$-*a* //delete the node “*a*” 13.  **end** 14.  Recalculate the load of all nodes 15.  **end** 16. **end** 17. **return** the minimum routing tree

First, for the convenience of the following description, each node in the minimum routing graph needs to be layered, that is, all nodes in the minimum routing graph that are one hop from the sink node are defined as the first level $l_{1}$, and the nodes in the two-hop distance from the sink node are defined as the second level $l_{2}$, and so on. In this way, similar operation is completed until finding the layer that contains the most peripheral nodes. Besides, it should be specified that one node only belongs to one layer. Finally, a hierarchical set *L* containing all nodes is obtained, namely:

(7) $$L=\{l_{1},l_{2},...,l_{max}\}$$

In the process of implementing the edge-cutting strategy, we start from the first layer $l_{1}$, and the load sizes of all nodes in the same layer are sorted. Here, it is divided into the following two cases:

- *Case 1* When there is only one node “*a*” with the least load in this layer, if the child nodes of node “*a*” have multiple candidate parent nodes, the link between the child nodes of node “*a*” and other candidate parent nodes are cut off. If the child nodes of node “*a*” only have one candidate parent node, the edge-cutting operation is not performed. Then, node “*a*” is removed from this layer and all nodes’ loads are updated.
- *Case 2* When there are multiple nodes with the least load simultaneously, the smaller the load product of all the nodes on the shortest path which is from this node to the sink, the larger the residual energy of this node, thus the smaller the amount of data transfer undertaken by this path. That is, the advantage of this node as next hop is greater. Here the “node product”, expressed as:
  (8) $$LCN(i)=\frac{\prod_{\zeta \in path_{tf}^{is}-sink}LN(\zeta )}{E_{i}}$$
  where $path_{tf}^{is}$ is the final shortest path left by the node i after the previous edge-cutting operation, and ζ represents all nodes on this final shortest path except the sink node.

Based on Equation (8), the node with smallest node product is set to “*a*”, if the child nodes of node “*a*” have multiple candidate parent nodes, then the link between the child nodes and their other candidate parent nodes are cut off. If there is only one candidate parent node of node “*a*”, no edge trimming is performed. Then, node “*a*” is removed from the layer and all nodes loads are updated.

The same operation is used to traverse other nodes in the same layer until all nodes in this layer complete the edge-cutting. Meanwhile, this strategy will be performed on other layers until all layers complete the edge-cutting. 

**Figure 2:**
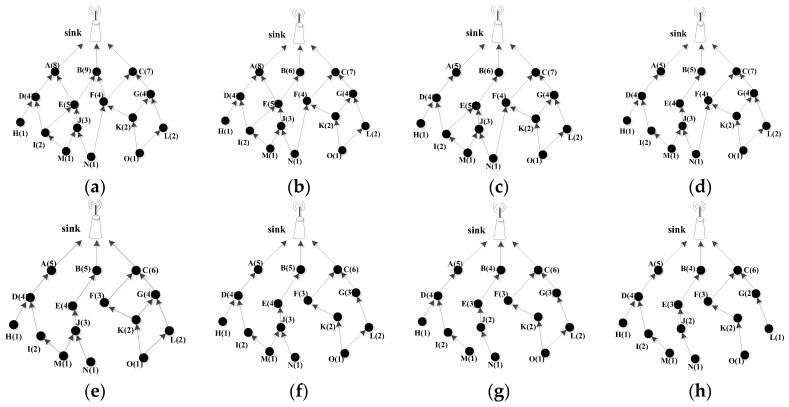
Example of the edge-cutting process. (Note: the value in parentheses indicates the corresponding node load, (**a**) is the minimum routing graph, (**h**) is the minimum routing tree, (**a**–**h**) show the edge-cutting process from the minimum routing graph to the minimum routing tree).

Here, in order to explain the working process of edge-cutting strategy more clearly, this paper takes a simple network topology in Figure 2 as an example. The details are as follows: 

The minimum routing graph including all nodes in the network shown in Figure 2a is generated according to the link cost. Firstly, the load of first layer node A, B, and C can be calculated, at this time, it shows that node C has the smallest load, so the link FB is cut off and the loads of other nodes are updated, and Figure 2b is obtained. In Figure 2b, node B becomes the node with the smallest load in first layer, so the link EA needs to be cut off and the load of each node is updated. At this point, the edge-cutting of all the nodes in the first layer is completed, and Figure 2c is obtained.

In Figure 2c, the second layer consists of four nodes, namely D, E, F, and G. The link IE is firstly cut off and the node loads are updated, then we can get the Figure 2d. Here, considering that the loads of node D, E, F, and G are the same and the smallest, according to above Case 1, node D is the only parent of its child node H and I, so the node D doesn’t need to perform edge-cutting processing. Then we need to cut off the link NF since the node product of node E is the smallest and update all node loads according to the foregoing case 2. Hence, Figure 2e is obtained. Moreover, the load of node F is smaller than that of node G, so the link KG is cut off and all node loads are updated. At this point, all nodes in the second layer have completed the edge-cutting, and Figure 2f can be obtained. 

In Figure 2f, according to the Case 1, node H does not need to implement the edge-cutting, and the link MJ need to be cut off and all node loads are updated to obtain Figure 2g. At this point, the node H, I, and J do not need to perform the edge cutting. According to the Case 2, the node product LCN(K) is less than LCN(L) after calculation, so the link OL is cut off and all node loads are updated. Finally, the minimum routing tree with minimum link cost and maximum load balance as shown in Figure 2h is obtained.

## 4. Simulation Results and Analysis

In order to verify the effectiveness of our algorithm, this paper adopts MATLAB to simulate, and conducts comparative analysis with the FAF-EBRM and FLEOR algorithms. In addition, five performance parameters will be used in the following paper: average energy consumption (AEC), average hop count (AH), packet loss rate (PLR), energy variance (EV), and node survival rate (NSR). Among them, AEC represents the average energy consumption of all nodes in each data transmission round, which measures the utilization efficiency of network energy. AH can reflect the transmission delay of data in the network, and the smaller the average hop count is, the faster data can be transmitted to sink. PLR can reflect the network’s ability in congestion avoidance. EV can give expression to the difference of energy residual value of each node, that is, the smaller the value of EV, the smaller the curve fluctuation is, thus the more balanced energy of the network. NSR is used to indicate the network lifetime and the effectiveness of energy utilization. The steeper the slope of the curve from the death of first node to the death of all nodes, the more concentrated time for the nodes to run out of energy, and the more balanced energy in the network. In addition, in order to avoid the contingency of the experimental results, we repeat each experiment many times to get an average.

The specific simulation parameters are listed in Table 1.

### 4.1. The Impact of Parameter β on Network Performance

In order to compare the influence of different parameters on the algorithm itself, in the simulation, we value the β under different network sizes, and the interval and step size are [0, 1] and 0.2 respectively.

(1) Impact of parameter β on average hops under different network sizes

**Figure 3:**
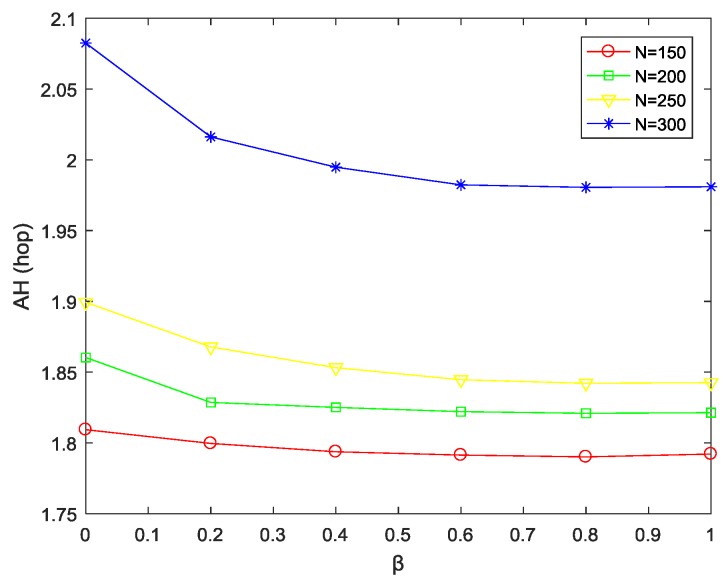
AH varying with β at different network sizes.

It can be seen from Figure 3 that, under the same network size, when the parameter β increases from 0 to 0.6, the average hops gradually decreases. This is because as β increases, the forward energy consumption of node *j* has an increasing influence on the selection of the next hop, that is, the source node will focus on selecting the forward neighbor node closer to the sink as next hop, thus the number of path hops is gradually reduced. When β is in [0.6, 1], the path hops tend to be stable as β increases further. In addition, when β remains the same, as the network size expands, the node distribution becomes denser, so that the average hops increases continuously.

(2) Impact of parameter β on average energy consumption under different network sizes

**Figure 4:**
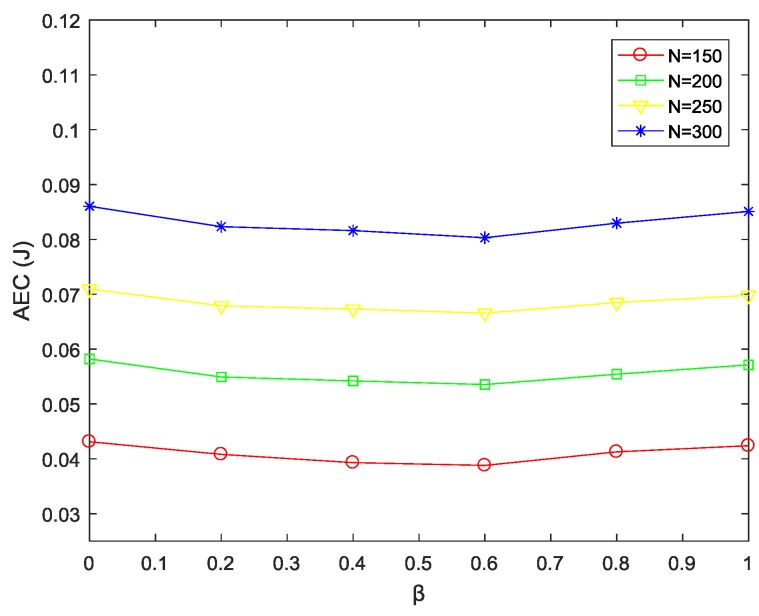
AEC varying with β at different network sizes.

As shown in Figure 4, when β is the same, as the network size increases, the average hops increases gradually (Figure 3), so the energy consumed on the path increases, resulting in the increasing of the average energy consumption of the entire network. When the network size remains the same and β increases in [0, 0.6], the average energy consumption gradually decreases. This is because as β increases, on the one hand, the hops have an increasing influence on the link cost in Equation (4). On the other hand, as shown in Figure 3, the average hops gradually decreases in [0, 0.6]. Thus, the overall energy consumption of the network will gradually decrease. When β is in the range of [0.6, 1], the average hops basically remains the same, but since the selection of the next hop neglects the influence of single-hop energy consumption, the average network energy consumption has a slowly increase in this range. 

(3) Impact of parameter β on packets loss rate under different network sizes 

**Figure 5:**
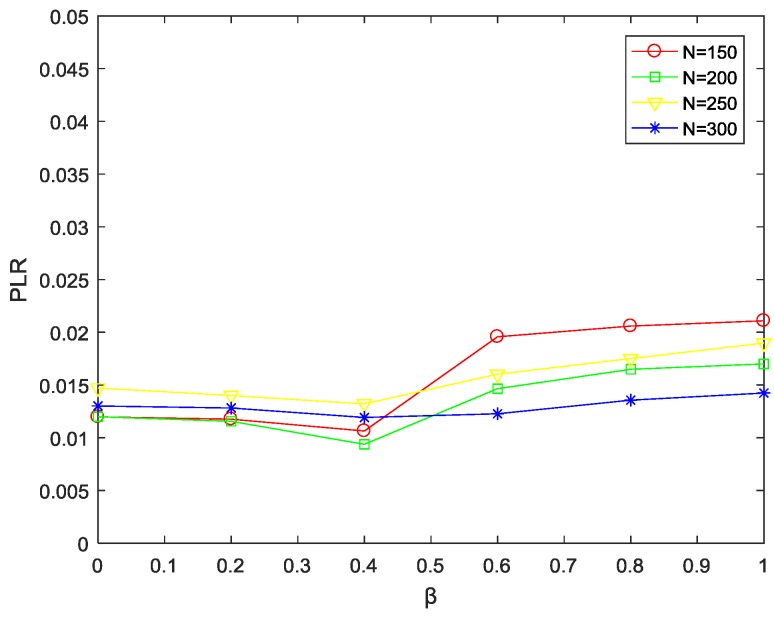
PLR varying with parameter β at different network sizes.

Figure 5 shows the trend of packet loss rate PLR with parameter β at different network sizes. When the network size is the same, the packet loss rate decreases as β increases in [0, 0.4], this is because the value of β is small at this time, and the hops do not dominate the selection of the path.

Meanwhile, after using the edge-cutting strategy, the load of each node in the minimum routing graph is balanced, thereby reducing the packet loss rate of the node. However, as β continues to increase, the node near the sink will be the priority node, that is, the path with less hops is selected to forward the data as much as possible, which will cause the nodes close to the sink to be over-selected and exceed the their capacity, resulting in packet loss. Then, the packet loss rate will increase slightly and tend to be stable. When β remains the same, as the network size *N* increases, the forward neighbor nodes in the inner layer gradually increase. By utilizing the edge-cutting strategy, excess load will be shared to other nodes with fewer loads in advance, thus effectively reducing the packet loss rate.

### 4.2. Comparison with Other Algorithms

**Figure 6:**
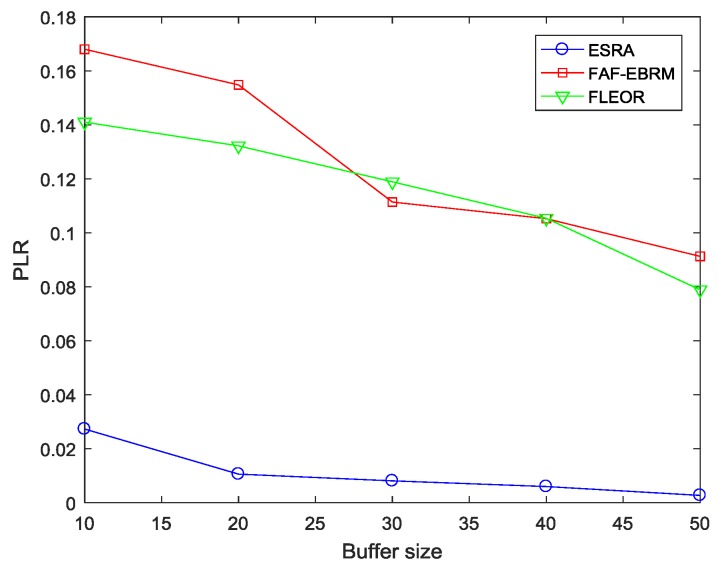
The PLR comparisons with varying buffer size.

Firstly, in order to validate the proposed protocol and perform a fair comparison, we take the change of buffer size as an example and carry out experimental simulation in this section. Furthermore, as the buffer size increases, the ability of the node to accommodate the data packet is improved, and the degree of congestion in the area can be alleviated to some extent, so the packet loss rate will be reduced accordingly. Here, Figure 6 shows the trend of packet loss rate with varying buffer size. It can be seen that the packet loss rate does decrease with the increase of the buffer size, and our algorithm, that is ESRA, shows obvious advantages.

Then we analyze the time complexity of these three algorithms. In our proposed ESRA, an initial shortest path from each source node to the sink can be calculated according to the Dijkstra algorithm, and the required time complexity is $O(N^{2})$. Then, all the paths that adjacent to their initial shortest path of the same source node are calculated, and the paths are sorted according to the weights by the merge sorting to obtain the shortest path set, so the time complexity is O(N•logN). The next step is to layer all nodes, and the required time complexity is O(N). After that, we need sort the loads of all nodes and implement the edge-cutting strategy, the time complexity of this step is O(N•logN). In general, the time complexity of our proposed algorithm in a worst-case scenario is $O(N^{2})$. As for FLEOR and FAF-EBRM, after getting different values by considering some attributes of the forward neighbor nodes, the neighbor node with the largest value is selected as the next hop by sorting, namely, the time complexity required of both algorithms is O(N•logN).

From the above analysis, it can be found that the computation of our proposed ESRA is slightly larger than FAF-EBRM and FLEOR, but it shows obvious advantages in terms of network performance according to the subsequent comparative analysis, which is detailed as following sections.

#### 4.2.1. Average Hops and Average Energy Consumption

**Figure 7:**
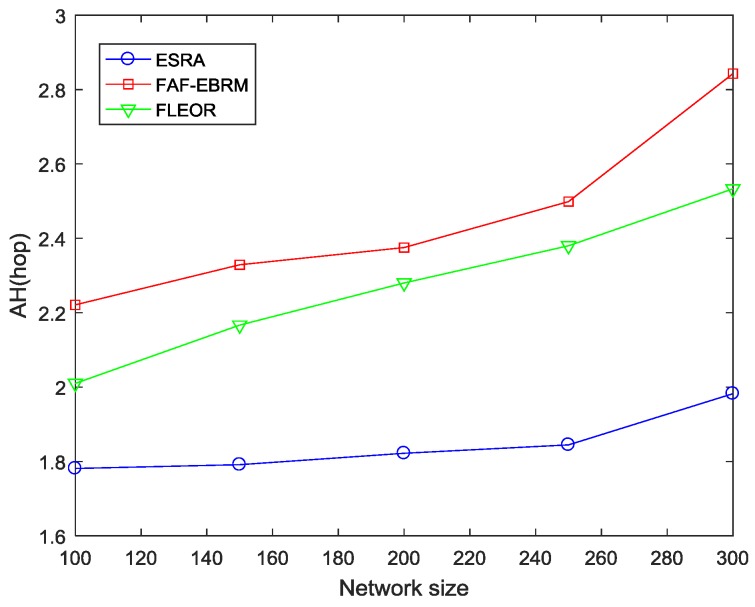
The AH comparisons with varying network size.

**Figure 8:**
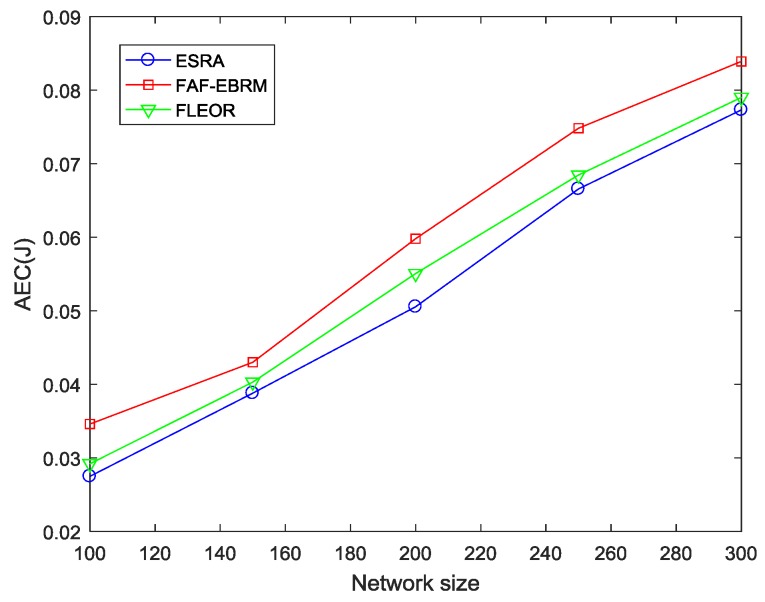
The AEC comparisons with varying network size.

Figure 7 and Figure 8 show the average hops and average energy consumption of the network at different network sizes. It can be seen from the figures that the two have similar trends since hops can reflect the energy consumption of the path in some way. In addition, the FAF-EBRM algorithm has the highest average hops and energy consumption. This is because FAF-EBRM detours for selecting nodes with higher energy as the next hop when routing, resulting in higher hop count and energy consumption. The average hops of FLEOR relative to FAF-EBRM is smaller, this is because FLEOR considering the selected path as close as possible to the shortest path, thus reducing the data forwarding times and average energy consumption. As for the ESRA algorithm of this paper, when β is taken as 0.4, the single-hop energy consumption has a greater influence on the selection of next hop. Simultaneously, as shown in Figure 7, the ESRA algorithm can always maintain the smallest average hops, therefore, compared with FLEOR and FAF-EBRM, our algorithm can effectively reduce the path energy consumption of the whole network, and transfer data from source nodes to the sink node faster. 

#### 4.2.2. Traffic Balance and Energy Balance

Usually, the node load affects network performance in two ways. On the one hand, the node load can reflect the amount of traffic that is aggregated to the node at some point. The larger the load, the more data need to be transferred. On the other hand, the number of the loads also determines the residual energy of node to a certain extent, that is, the larger the load, the less energy the node has due to the greater transmission energy consumption. In addition, the more balanced the energy consumption between nodes, the higher the node survival rate and the longer the network lifetime. Therefore, on behalf of measuring the balance of the network, we consider both traffic balance and energy balance in this section.

(1) Traffic Balance 

**Figure 9:**
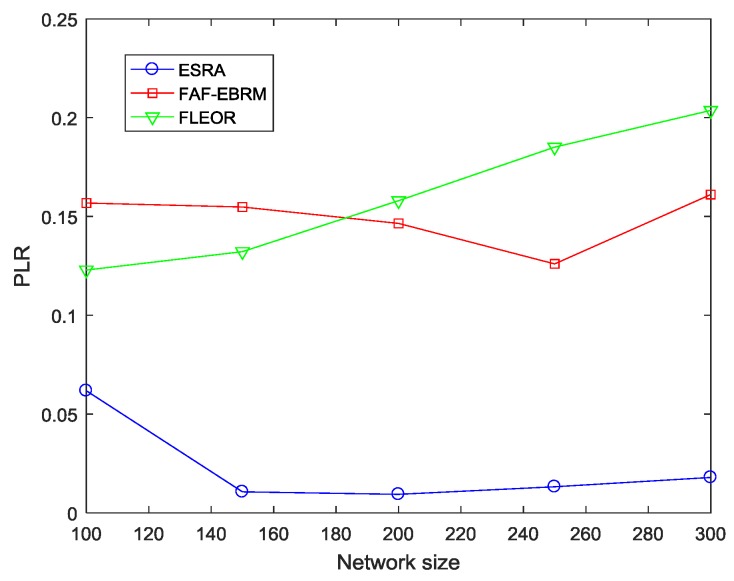
The PLR comparisons with varying network size.

Figure 9 shows the different packet loss rates of different algorithms at different network sizes. It can be seen that compared with the other two algorithms, the packet loss rate of ESRA algorithm is always stable at a lower value as the network size changes. Obviously, this is because FAF-EBRM and FLEOR do not take into account the queue size of the nodes. A large amount of data are concentrated in some “hot spots” at the same time, causing nodes in this area to exceed their load capacity due to excessive data reception, thus a large amount of data are discarded. On the contrary, our algorithm adopts the edge-cutting strategy to balance the node load. In the minimum routing graph, the load is updated in real time, and the candidate parent nodes with smaller load are dynamically selected as the forwarding node, which alleviates the congestion degree, so the network traffic is relatively balanced. 

(2) Energy Balance 

**Figure 10:**
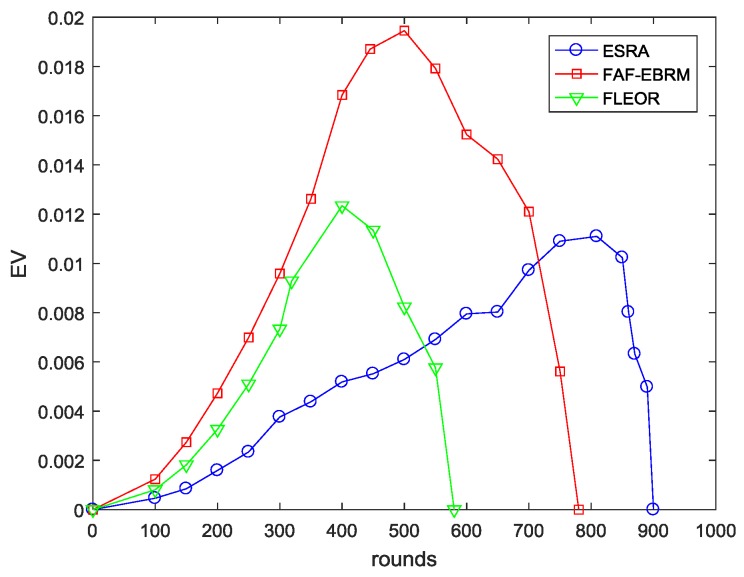
The EV comparisons with varying rounds.

**Figure 11:**
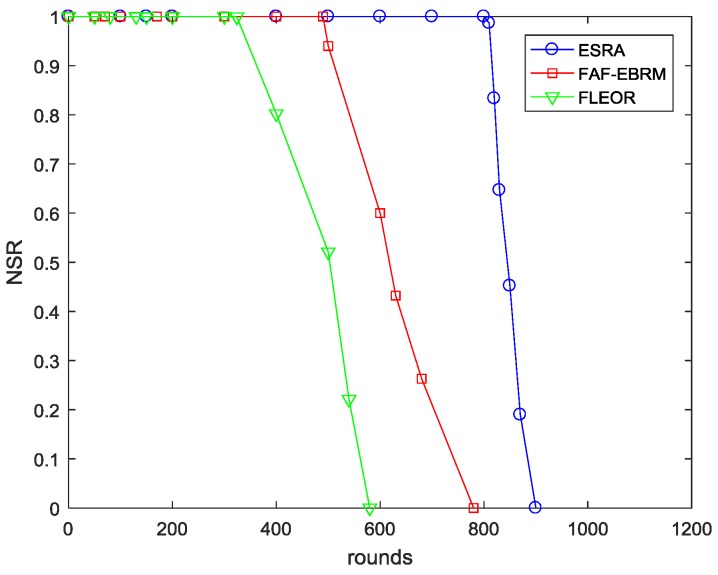
The NSR comparisons with varying rounds.

For the three different algorithms, the comparison of the energy variance EV and the node survival rate NSR are given in Figure 10 and Figure 11, respectively. Among them, the EV curve of the FAF-EBRM algorithm has the highest volatility, and the energy balance is worse than the other two algorithms. The energy balance of FLEOR is improved compared with FAF-EBRM, this is because FLEOR considers the energy balance of the forward neighbor, and prefers the forward neighbor with larger residual energy as next hop. However, the network lifetime of FLEOR is shorter than FAF-EBRM, and the first node death appears in around 320 rounds. In this paper, the ESRA algorithm can sense the node loads in some way, which can optimize the routing of the next data round and rebalance the energy distribution. Therefore, the EV curve has the smallest fluctuation and the node survival rate is maintained at a highest level at all times. Simultaneously, according to the rounds of the first dead node, its network lifetime is about 80% and 150% longer than FAF-EBRM and FLEOR respectively, which means that the network can maintain a longer effective working time. In addition, when all nodes in FAF-EBRM have died, ESRA has not yet appeared the death of first node, showing a good network performance.

## 5. Conclusions

In order to reduce the transmission energy consumption, and balance the node load to achieve the purpose of extending the lifetime of wireless sensor networks, this paper proposes an equilibrium strategy based routing optimization algorithm for wireless sensor networks (ESRA). Considering the residual energy, single-hop energy consumption and path hops, the algorithm firstly establishes a minimum routing graph. Then, an edge-cutting strategy is adopted to balance the node load on the topology of the minimum routing graph. The simulation results show that our proposed algorithm can prolong the network lifetime, balance the node traffic and residual energy, and also reduce the transmission delay and packet loss rate. In the future work, we will focus on how to jointly utilize the energy-harvesting technology and routing optimization algorithm to enhance the network performance.

## Figures and Tables

**Table 1:** Simulation Parameters.

Definition	Value
Simulation area	100 × 100 m^2^
Network size	150~300
Maximum communication range	30 m
Packets size	1024 bits
Buffer size	20 packets
Sink	(50, 50)
Data generation rate	1024 bits/round
